# Economic evaluation of health consequences of prenatal methylmercury exposure in France

**DOI:** 10.1186/1476-069X-11-53

**Published:** 2012-08-10

**Authors:** Céline Pichery, Martine Bellanger, Denis Zmirou-Navier, Nadine Fréry, Sylvaine Cordier, Anne Roue-LeGall, Philippe Hartemann, Philippe Grandjean

**Affiliations:** 1 EHESP School of Public Health, Rennes Cedex 35043, France; 2 Lorraine University Medical School, Public Health department, Vandoeuvre-les-Nancy cedex, France; 3 INSERM U 1085-IRSET (Institut de Recherche Santé Environnement Travail), Rennes 1 University, Rennes cedex 35042, France; 4 Institut de veille sanitaire, Département Santé Environnement, Saint Maurice cedex 94415, France; 5 INSERM U 954 "Nutrition, genetics and environmental risks”, Medical School, Vandoeuvre-les-Nancy cedex 54505, France; 6 Institute of Public Health, University of Southern Denmark,Odense DK-5000, Denmark; 7 Department of Environmental Health, Harvard School of Public Health, Boston, MA 02215, USA

**Keywords:** Economic evaluation, Methylmercury, Prenatal exposure, Neurodevelopmental deficits

## Abstract

**Background:**

Evidence of a dose–response relationship between prenatal exposure to methylmercury (MeHg) and neurodevelopmental consequences in terms of IQ reduction, makes it possible to evaluate the economic consequences of MeHg exposures.

**Objective:**

To perform an economic evaluation of annual national benefits of reduction of the prenatal MeHg exposure in France.

**Methods:**

We used data on hair-Hg concentrations in French women of childbearing age (18–45 years) from a national sample of 126 women and from two studies conducted in coastal regions (n = 161and n = 503). A linear dose response function with a slope of 0.465 IQ point reduction per μg/g increase in hair-Hg concentration was used, along with a log transformation of the exposure scale, where a doubling of exposure was associated with a loss of 1.5 IQ points. The costs calculations utilized an updated estimate of €_2008_ 17,363 per IQ point decrement, with three hypothetical exposure cut-off points (hair-Hg of 0.58, 1.0, and 2.5 μg/g).

**Results:**

Because of higher exposure levels of women in coastal communities, the annual economic impacts based on these data were greater than those using the national data, i.e. € 1.62 billion (national), and € 3.02 billion and € 2.51 billion (regional), respectively, with the linear model, and € 5.46 billion (national), and € 9.13 billion and € 8.17 billion (regional), with the log model, for exposures above 0.58 μg/g.

**Conclusions:**

These results emphasize that efforts to reduce MeHg exposures would have high social benefits by preventing the serious and lifelong consequences of neurodevelopmental deficits in children.

## Background

Human exposure to methylmercury (MeHg) occurs primarily through ingestion of seafood and freshwater fish [1]. Due to biomagnification in food chains, relatively high MeHg concentrations occur in piscivorous marine species [2] and may exceed the highest recommended limit [3], while smaller non-predatory species, such as herring or sardine, contain concentrations of one-tenth of this limit or even less [3]. Methylation, bioaccumulation through food chains, and human intake levels are difficult to model [1]. Thus, risk assessment must rely on biomarkers of total human uptakes.

Once absorbed, MeHg acts as a developmental neurotoxicant [4-7]. As the critical effect is considered to be developmental brain toxicity [8,9], MeHg intake by pregnant women is of primary concern [10]. In the 1990s, results emerged from three large epidemiologic studies in New Zealand, the Faroe Islands and the Seychelles Islands [6,11-14]. The first two concluded that chronic low-dose prenatal MeHg exposure from maternal consumption of fish was associated with subtle end points of neurotoxicity in children [15]. Support for the notion of seafood-mediated MeHg neurotoxicity later emerged also from the Seychelles [16]. In further research, Faroes investigators provided extended evidence of a dose–response relationship between prenatal MeHg exposure and lasting neurodevelopmental deficits [15,17,18]. Subsequently, epidemiological studies in French Guiana [19-21] and in other parts of the world [22-24] showed the effects of MeHg on childhood neurodevelopmental disorders. This research has prompted further studies focused on French populations, especially in coastal regions of western France [3,25].

Reducing human exposure to anthropogenic mercury is both a public health priority and an economic challenge, and controversies persist in both research interpretation and policy decisions [10]. The consequences of MeHg contamination, similar to those observed for lead (Pb) exposure in children, include a loss in Intelligence Quotient (IQ), with associated lower school performance and educational attainment, thus leading to long-term impacts on societal benefits of pollution abatement [26,27]. The economic impacts caused by MeHg on humans have been assessed in the United States, through the studies by Rice et al. [28,29] and publications from the US Environmental Protection Agency (EPA) [30], and the study of Griffiths et al. [31]. Although these calculations have been extrapolated to global estimates [32,33], few economic evaluations have been performed in Europe [34,35]. To extend and update the international assessments of economic impacts of MeHg exposure and to utilize biomonitoring data, the present study aims to assess the economic consequences of MeHg-associated neurotoxicity, using exposure data from French studies. As biomonitoring results become available from other countries, additional national estimates can be made using the same methodology.

The economic assessment requires toxicological and epidemiological assumptions. Hg concentrations in hair and in umbilical cord blood have been used as biomarkers to ascertain prenatal exposure to MeHg, although both exposure indicators are associated with some imprecision [36]**.** In selecting the dose–response function (DRF), a major difficulty arises when deciding on the MeHg dose metric [15]. While a linear model is attractive, it does not provide the best statistical fit to the data [18]. Studies that used a log transformed exposure scale assume that each doubling of exposure causes the same deficit. In the absence of a clear threshold, an additional decision has to be made in regard to a toxicological reference value, so that the epidemiological findings are translated into a “cut-off point”, below which only negligible adverse effects exist. Using both a linear and a logarithmic dose–response curve (DRC), we provide estimates of the economic consequences of prenatal MeHg exposure for three different such cut-off points as a basis for development of public policies to prevent MeHg exposure at national and international levels.

## Methods

### Data sources

#### Three samplings of maternal hair

The first source of data is the 2006–2007 French national survey on nutrition and health ENNS (Etude Nationale Nutrition Santé) run by the French Institute for Public Health Surveillance (InVS). We used a national sub-sample of 18–45 year old women (*n* =126) representing the population of childbearing age. The geometric mean of hair-Hg concentrations was 0.53 μg/g (interquartile range 0.37-0.82 μg/g; full range 0.073-5.1 μg/g) [37].

The second source of data is the regional 2002–2006 PELAGIE cohort study from Brittany, the most western region of France, partly surrounded by the Atlantic Ocean, carried out by the National Institute of Health and Medical Research (INSERM U1085) to explore the role of environmental pollutants on pregnancy and delivery outcomes, and on children’s health and development [25]. The PELAGIE cohort includes 3421 pregnant women enrolled in early pregnancy by medical practitioners in three districts of Brittany. A sub-cohort of 601 women was randomly selected for biomarker determination, including mercury from 503 (84%) maternal hair samples collected at delivery. The geometric mean of hair Hg concentrations was 0.62 μg/g (interquartile range 0.40-0.94 μg/g; full range 0.06-3.42 μg/g).

The third source of data is a 2005–2006 study from the National Institute of Agricultural Research (INRA) [3]. In this study, hair Hg levels were clustered in relation to fish consumption of pregnant women admitted in three hospitals in the Loire-Atlantique coastal district. Two hospitals were located in Nantes, the regional capital, and its suburb, and one in Saint-Nazaire. The frequency of fish consumption is higher in this region than in other French regions more distant from the ocean [38]. Fish consumption and hair mercury concentrations were assessed during two visits at 12 and 32 weeks of pregnancy (*n* =161 for the first visit; *n* =137 for the second). The more complete first sample showed a geometric mean hair-Hg concentration of 0.67 μg/g (interquartile range 0.42-0.94 μg/g; full range 0.00-3.66 μg/g).

#### Reference values for hair-mercury and conversion into cord blood concentrations

Different toxicological reference values for neurotoxicity have been recommended for setting exposure limits. Thus, the Joint FAO/WHO Expert Committee on Food Additives (JECFA) recommends MeHg doses not to exceed 0.23 μg/kg body weight per day (bw/day), corresponding to 2.5 μg/g hair, above which there may be a risk for children, especially through exposure of pregnant or lactating women [39]. The U.S. EPA developed a MeHg Reference Dose (RfD) which is 0.1 μg/kg bw/day, corresponding to 1 μg/g hair [1]. We also considered a third value based on updated findings from the prospective studies in the Faroes [36], which resulted in an exposure limit about 50% below the level recommended by the U.S. National Research Council (NRC), corresponding to 0.58 μg/g hair, the lowest of the three reference values. Below, these three values will be used as three hypothetical cut-off points. MeHg exposures at these levels are considered to be negligible and acceptable, but they do not necessarily represent a level at which there is no effect on neurodevelopment [15].

### The dose–response function for IQ losses

Several possible linear and logarithmic dose–response models have been proposed to represent the relationship between Hg exposure and the neurodevelopmental outcomes. Based on the Faroe Islands data, the NRC recommended a linear dose–response model [15]. The Faroes’ investigators showed that a logarithmic model provided a better fit to the data than the linear one, although the difference between the two was barely statistically significant [18]. Accordingly, we used both the linear DRF model, and the log DRF model.

For the linear model, a 1 μg/L increase of the cord blood Hg concentration is associated with an average adverse impact on IQ of 0.093 IQ point of the standard deviation (SD), which is 15, thus estimated at 0.465 IQ points [40], assuming that the ratio between mercury in hair and in cord blood is 200. These values derive from the Budtz-Jørgensen report [18] and pertain to a range of neuropsychological tests and subtests administered in the Faroe Islands study when the children were assessed at age 7 years, including IQ subtests.

Hence, assuming a linear DRF and a central estimate of the slope of 0.465 IQ points per μg/g hair increase, we computed IQ decrements above the three hypothetical cut-off points defined above. Losses of IQ were estimated for the following concentration ranges: [0.58 μg/g – 1.0 μg/g], [1.0 μg/g −2.5 μg/g] and ≥2.5 μg/g, based on

(1) y'=0.465x+b

Where *y*’ denotes the change in IQ point and *x* is the hair-Hg concentration, and *b* the intercept specific for each cut-off point. Thus, *y*’ equals 0 at each hypothesized cut-off point:

(2) $$y{'}_{0.58\mu g/g}=0.465\;x-0.27$$

(3) $$y{'}_{1\mu g/g}=0.465x-0.465$$

(4) $$y{'}_{2.5\mu g/g}=0.465\;x-1.162$$

**Figure 1:**
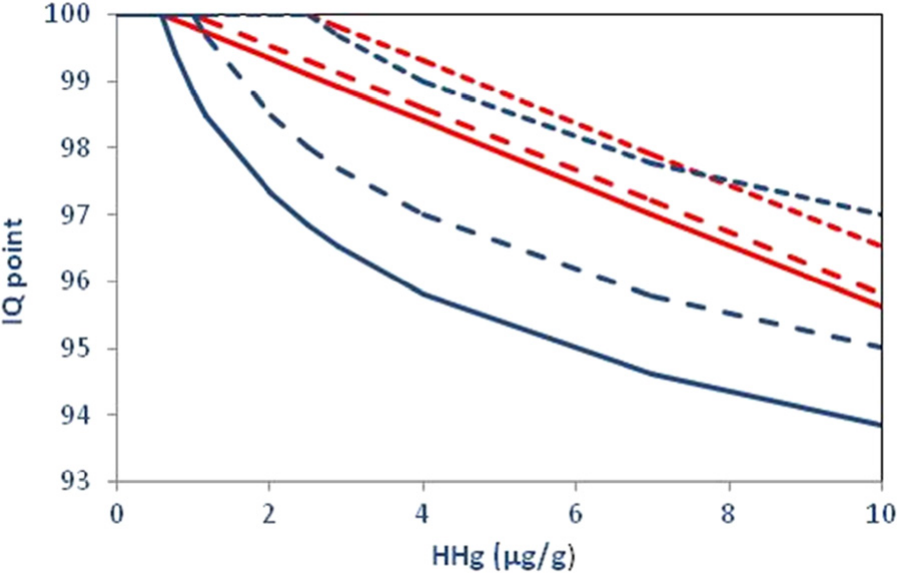
**Loss of IQ points according to HHg concentrations based on linear and log models.** Figure 1 displays the comparison of IQ decrements associated with HHg concentrations based on a linear model in red lines and a logarithm model in blue lines. In the first, estimates of IQ point loss are presented for the three cut–off points 0.58, 1.0 and 2.5 μg/g and for the maximum (i.e. the Percentile P99.5, extreme value excluded) for the three samples. In the log model, IQ point losses are given above 0.58 μg/g. And, we assumed no IQ loss below the three cut-off points.

We assumed a stable diet of infants and mothers, so that any detailed time distribution of the sensitivity to Hg does not matter for the calculation of impacts [32]. Consequently, we considered that the DRF slope (0.465 IQ points per μg/g hair) in equation (1) represents the lifetime neurodevelopmental impairment experienced by a child whose mother has been exposed to a continuous Hg dose indicated by the hair-Hg concentration (HHg) measured. Thus, the lifetime impact on a child exposed above the three selected cut-off points, was estimated according to equations (2), (3) and (4) (Figure 1). In the linear model, we selected the median of each of the lowest intervals [0.58-1 μg/g], [1–2.5 μg/g] to represent all subjects within the interval, i.e. 0.79 μg/g and 1.75 μg/g. For the national sample, 2.62 μg/g was considered to represent all subjects with results above 2.5 μg/g based on the Percentile P99.5, while excluding the extreme value (2.74 μg/g). Similarly, the mid-points for the highest exposure group were 2.76 μg/g for the Brittany sample and 3.08 μg/g for the Loire Atlantique sample.

For the log model, the Faroes data suggested that the most sensitive brain functions showed a delay in development of 1.5–2 months at age 7 years associated with each doubling of the prenatal MeHg exposure. This delay corresponded to about 10% of the SD for these tests, which would correspond to about 1.5 IQ points [41]. So the equation for IQ loss above the lowest cut-off point is the following:

(5) $$IQ=IQbaseline-\alpha *(\log2\left(HHg/0.58\right)$$

Where IQ baseline is the IQ with 100 points, α is 1.5 IQ points, HHg is the hair–Hg concentration (log2 transformed), and 0.58 (μg/g) the cut-off point (see Figure 1).

For the interval [0.58-1 μg/g], the mid-point on the log scale is chosen as the representative exposure for all subjects within this interval. The mid-point is 0.76 [antilog (−0.12)], which corresponds to an increase of HHg by 31% above the 0.58 μg/g cut-off. As a doubling would result in an IQ loss of 1.5 points, the increase by 31% corresponds to an IQ loss of slightly more than 0.5 points. This is used as the average IQ loss for all subjects within this interval. For the interval [1–2.5 μg/g], the value of 1.16 is chosen as representative for the subjects within this interval due to the skewed distribution of HHg with most subjects much closer to 1 than to 2.5 μg/g. The value of 1.16 corresponds to a doubling of the 1 cut-off level, thus to an average IQ loss of 1.5 points. Similarly, subjects above the 2.5 cut-off were assigned an average of 2.9 μg/g, i.e. 5 times the cut-off or 2.32 doublings, which correspond to a loss of 1.5*2.32 = 3.5 points.

### Annual benefits of exposure reduction

As explained above, the effects of prenatal MeHg toxicity on children can be considered to be similar to those of developmental lead exposure. Both exposures are associated with a reduced IQ, which in turn has a negative impact on the social benefits. According to an impact evaluation applied to childhood lead exposure [26], the major component of the social costs incurred by an IQ reduction is loss of productivity and thus a lower earning potential. In the present study, the economic consequence of MeHg prenatal exposure is assessed for a birth cohort of children born to women of childbearing age (18–45 years) and valued as the lifetime earning loss per person and extrapolated to the French national birth cohort of 834,000 children born in 2008 [42]. That year was found to be the closest to the time during which the exposure data had been collected. We assumed singleton births only, so that the number of women was equal to the cohort size.

#### Health impact: MeHg lifetime impact on the exposed population

In the national sample, the Brittany study and the Loire Atlantique study, respectively, the exposure levels were based on the percentage of women with hair Hg concentrations within the 0.58 μg/g - 1 μg/g, 1 μg/L - 2.5 μg/g and ≥2.5 μg/g ranges. For the three studies, these were 43.8%, 14.5%. and 0.6% (national); 55%, 33%, and 1.2% (Brittany); and 60%, 22.5%, and 1.9% (Loire Atlantique). Lastly, we applied those percentages to the 2008 cohort assuming the three sample distributions measured the *lifetime impacts*, i.e. the effects of MeHg in terms of IQ points permanently lost. Although some compensation may be possible over time, current evidence suggests that MeHg-linked cognitive deficits are lasting [17]. Irrespective of future exposure reductions a child whose IQ has been impaired due to early life exposure will never recover from a retardation that is irreversible.

#### Economic impact: Benefits of reduction of the MeHg exposure

The estimated individual benefits are the avoided lifetime costs. They originate from the figure of €_2008_17, 363 per IQ point loss that we published recently for Pb intoxication [26], the most recent value available. We computed the MeHg-related avoided cost for an IQ point decrement for an individual i, denoted B_i_, as follows:

(6) $$B_{i}=NIQ_{i}\times €\;17,363$$

Where $NIQ_{i}\;$is the number of IQ points loss for subject i.

The population benefits of reducing mercury exposure were estimated within the three concentration ranges*:* [0.58- 1], [1–2.5] and ≥2.5 μg/g denoted B_[range]_*,* as follows:

(7) $$B_{\left[range\right]}=\sum_{i}B_{i}$$

where $\sum_{i}$denotes the sum of all individual benefits within a given exposure range.

Lastly, the total population benefits (TB) are cumulative, thus being the sum of the B_range_ values within each segment of the corresponding distribution: denoted *TB*_*0.58*_, *TB*_*1*_ and *TB*_*2.5*_, respectively, according to the following equations:

(8) $$TB_{0.58}=B_{\left[0.58-1\right]}+B_{\left[1-2.5\right]}+B_{\left[2.5-\max\right]}$$

(9) $$TB_{1}=B_{\left[1-2.5\right]}+B_{\left[2.5-\max\right]}$$

(10) $$TB_{2.5}=B_{\left[2.5-\max\right]}$$

The estimated benefits B_i_, B_[range]_ and TB based on lost earnings are valuated at their present value since they correspond to current exposure.

## Results

As shown in Table 1, the three study populations exhibit different exposure distributions. While the exposure levels are the lowest in the national sample, the distribution is shifted towards higher values in Brittany and, even more so, in Loire Atlantique. Associations between IQ losses and HHg exposures assuming linear and logarithmic relationships are reported for the different distributions and scenarios in Figure 1.

**Table 1:** Number of children from the 2008 birth cohort exposed to different levels of MeHg based on HHg concentrations in three French population samples

**Distributions**	**HHg concentration ranges (μg/g)**	**Number of children (N)**	**(%)**
**National**	Hg < 0.58	126,101	26
	0.58 ≤ Hg < 1.0	244,529	50
	1.0 ≤ Hg < 2.5	115,926	24
	Hg ≥ 2.5	5,087	1
	*All*	*491,643*	*100.00*
**Brittany**	Hg < 0.58	285,228	38
	0.58 ≤ Hg < 1.0	183,480	25
	1.0 ≤ Hg < 2.5	265,212	36
	Hg ≥ 2.5	10,008	1
	*All*	*743,928*	*100.00*
**LA**	Hg < 0.58	203,496	29
	0.58 ≤ Hg < 1.0	312,750	44
	1.0 ≤ Hg < 2.5	171,804	24
	Hg ≥ 2.5	15,846	2
	*All*	*703,896*	*100.00*

The Table 1 presents the number of children from the 2008 birth cohort exposed to different levels of MeHg based on HHg concentrations in three French population samples. We noted that 58.95% of women of childbearing age corresponded to the national sample, 89% to the Brittany and 84% to the LA sample, respectively.

Table 2 and Table 3 present the IQ losses and the estimates of the economic impact for the linear model, expressed as benefits associated with Hg exposures above the three cut-off points for the 2008 birth cohort assuming exposure distributions based on the three study samples. Due to the differences in exposure, the greatest benefits would be achieved with the Brittany sample, should all values be reduced below 0.58 μg/g. The estimated median annual total benefits were € 1.62 billion, € 0.77 billion, and € 0.005 billion using the national sample, according to the three cut-off points (0.58, 1.0, and 2.5 μg/g). The corresponding values were € 3.02 billion, € 1.75 billion € and € 0.02 billion using the Brittany data, and € 2.51 billion, € 1.31 billion and € 0.07 billion from the Loire-Atlantique exposure distribution. If we had used a rounded cut-off level of exposure at 0.5 μg/g , the total benefits estimated for the national sample would have been about 17% higher than those estimated to be above the cut-off level of 0.58 μg/g.

**Table 2:** Estimated IQ losses for the selected HHg cut-off points range in the three samples with the linear model

		**National sample**			**Brittany sample**			**Loire Atlantique sample**		
**HHg concentrations ranges (μg/g)**		**[0.58;1.00]**	**[1.00;2.50]**	**[2.50;2.74]**	**[0.58;1.00]**	**[1.00;2.50]**	**[2.50;3.02]**	**[0.58;1.00]**	**[1.00;2.50]**	**[2.50;3.66]**
**Loss of IQ point**	**From 0.58**	0.20	0.89	1.00	0.20	0.89	1.13	0.20	0.89	1.43
	**From 1.00**		0.70	0.81		0.70	0.94		0.70	1.24
	**From 2.50**			0.11			0.24			0.54

The Table 2 presents the IQ losses, for the linear model, the upper bound value per segment from the three cut-off points (0.58, 1.0 and 2.5 μg/g) for the 2008 birth cohort assuming exposure distributions based on the three study samples.

**Table 3:** Estimated lifetime economic benefits of reducing MeHg exposure in the 2008 children’s cohort according to the three study samples with the linear model

		**National sample**			**Brittany sample**			**Loire Atlantique sample**		
**HHg concentrations ranges (μg/g)**		**[0.58;1.00]**	**[1.00;2.50]**	**[2.50;2.74]**	**[0.58;1.00]**	**[1.00;2.50]**	**[2.50;3.02]**	**[0.58;1.00]**	**[1.00;2.50]**	**[2.50;3.66]**
**B**_**i**_ **(€/individual)**	**From 0.58**	[0.00;3,473]	[3,473;15,453]	[15,453;17,439]	[0.00;3,473]	[3,473;15,453]	[15,453;19,668]	[0.00;3,473]	[3,473;15,453]	[15,453;24,829]
	**From 1.00**		[0.00;12,154]	[12,154;14,048]		[0.00;12,154]	[12,154;16,277]		[0.00;12,154]	[12,154;21,509]
	**From 2.50**			[0.00;1,938]			[0.00;4.166]			[0.00;9,398]
**B**_**[range]**_ **(€ billion)** (midpoint value)	**From 0.58**	0.45	1.09	0.08	0.33	2.51	0.18	0.56	1.63	0.32
	**From 1.00**		0.70	0.07		1.61	0.14		1.04	0.27
	**From 2.50**			0.005			0.02			0.07
**TB** **(€ billion)** (midpoint value)		**From 0.58**	**From 1.00**	**From 2.50**	**From 0.58**	**From 1.00**	**From 2.50**	**From 0.58**	**From 1.00**	**From 2.50**
		**1.62** [0.5;2.73]	**0.77** [0.06;1.48]	**0.005** [0.00;0.0098 ]	**3.02** [1.08;4.93 ]	**1.75** [0.12;3.39 ]	**0.02** [0.00;0.042 ]	**2.51** [0.84;4.13 ]	**1.31** [0.19;2.43 ]	**0.07** [0.00;0.15]

Table 3 presents the estimates of the economic impact for the linear model, expressed as individual benefits (B_i_), benefits per range (B_[range]_) and the total benefits for one year (TB), associated with Hg exposures from the three cut-off points for the 2008 birth cohort assuming exposure distributions based on the three study samples.

**Figure 2:**
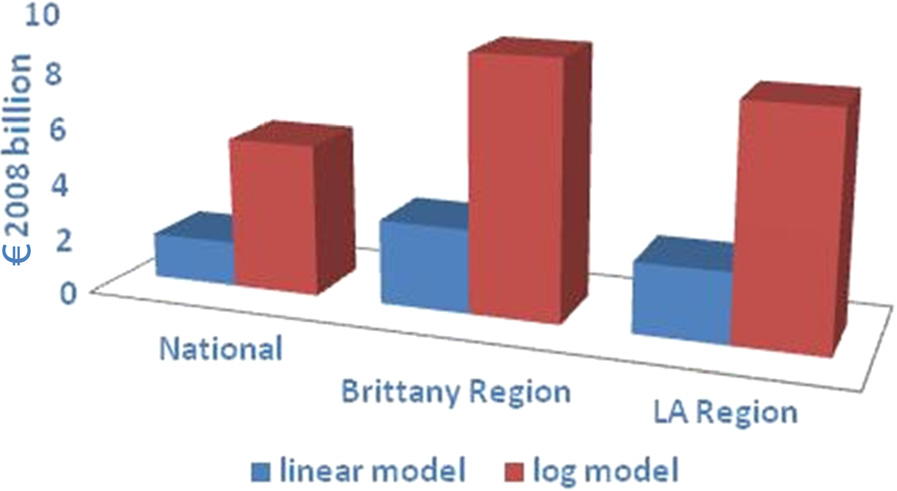
**Estimated annual benefits from MeHg reduction exposure above 0.58 μg/g, in the 2008 children cohort (in €**_**2008**_** Billion)**.

Table 4 presents the benefits associated with different levels of Hg exposure reductions by using the logarithm model. For all three samples, we used the same number of children per range for the both models (Table 1). The estimated total benefits were € 5.46 billion, € 9.13 billion, and € 8.17 billion based on the national, Brittany and Loire-Atlantique exposure distributions, respectively, above the 0.58 μg/g cut-off. Thus, the total benefits were estimated to be more than three times higher than those obtained from the linear model (Figure 2).

**Table 4:** Estimated lifetime economic benefits to reducing MeHg exposure in the 2008 children’s cohort according to the three study samples with the log model

	**National sample**			**Brittany sample**			**Loire Atlantique sample**		
**HHg concentrations ranges (μg/g)**	**[0.58;1.16]**	**[1.16;2.90]**	**[2.90;.Max ]**	**[0.58;1.16]**	**[1.16;2.90]**	**[2.90;.Max ]**	**[0.58;1.16]**	**[1.16;2.90]**	**[2.90;.Max ]**
**B**_**i**_**(from 0.58)** **(€/individual)**	8682	26134	60771	8682	26134	60771	8682	26134	60771
**B**_**[range[**_**(from 0.58)** **(€ billion)**	2.12	3.03	0.31	1.59	6.93	0.61	2.72	4.49	0.96
**TB (from 0.58)** **(€ billion)**	** 5.46**			** 9.13**			** 8.17**		

Table 4 presents the estimates of the economic impact for the logarithm model, expressed as individual benefits (B_i_), benefits per range (B_[range]_) and the total benefits for one year (TB), associated with Hg exposures from 0.58 μg/g for the 2008 birth cohort assuming exposure distributions based on the three study samples. Losses of IQ are 0.5, 1.5 and 3.5 IQ points for [0.58; 1.16], [1.16; 2.90] and [2.90; Max], respectively.

## Discussion

The aim of this article was to evaluate the economic impacts of neurotoxicity associated with prenatal MeHg exposure in France. Our estimations were carried out assuming a linear and a logarithmic relationship between Hg exposure and IQ losses [6,18], for three hypothetical cut-off points, 0.58 μg/g, 1.0 μg/g and 2.5 μg/g, respectively, based on three different evaluations [1], [36] and [39]. In agreement with European [43] recommendations, we do not pretend that any of the cut-off points are completely safe exposure levels, but merely represent hypothetical exposure levels below which adverse effects might be negligible. Three French data sets, i.e., national, Brittany and Loire Atlantique, enabled a characterization of the Hg exposure distribution from exposure biomarker results in women of childbearing age or pregnant women. Data were applied to the 2008 national birth cohort. We calculated the health impact (loss of IQ/individual) and the annual economic impact or benefits of Hg reduction in terms of personal avoided costs and for the entire cohort (Bi and TB, respectively), above each cut-off point.

These results highlight that prenatal MeHg exposure has serious impacts on the life-time productivity and on society due to adverse cognitive and associated economic consequences. Benefits were higher with the logarithmic than the linear model (see Figure 2), as the logarithmic DRF is steeper at low exposure levels that affect a larger proportion of children. The two regional exposure estimates, close to sea coasts, where marine food is more easily available, are higher than the national ones. This finding is in line with the French national statistics for fish consumption, which show that the population residing on the western coast of France (18% of the total population) consumes more of the total amount of fish available in the whole country for numerous species [38,44].

The results show that policies that aim to reduce childhood MeHg exposure would have large-scale social benefits. The focus on a child’s life-time earning loss is similar to the avoidable costs in relation to lead exposure reduction [26]. Other costs were ignored, such as direct medical costs linked to treatment or interventions for children with neurodevelopmental disorders. We also neglected indirect costs, such as those related to special education or additional years of schooling for children as a consequence of these disorders, as well as intangible costs. In addition, our study did not consider other avoided direct health care costs, such as those potentially related to the treatment of cardiovascular or neurodegenerative effects of MeHg exposure, which could be important for high fish consumers [5], but would be difficult to estimate.

Several uncertainties hamper accurate impact estimation. One concerns the slope of the dose–response relationship between maternal MeHg exposure and loss of IQ points. Our first estimates used an average mean of 0.465 IQ point loss per μg/g in HHg. The lower boundary (0.295 IQ point loss per μg/g) or the upper one (0.62 IQ point loss per μg/g) can be also used to obtain minimal or conservative estimates. We used both the linear and the log DRF, as the latter scale showed a better fit, suggesting that a doubling of exposure is associated with a loss of 1.5 IQ points [41]. This slope is of course also uncertain.

Three large-scale prospective epidemiologic studies investigated children who experienced MeHg exposures *in utero* at concentrations relevant: the Faroes study [6], the New Zealand study [11,12], and the Seychelles study [13,14]. These studies provided evidence of a dose–response relationship between concentrations of MeHg and neuro-developmental disorders among children [9]. As also decided by the NRC [15], we relied on the Faroes study as the most extensive data base that was only minimally affected by confounding. This way, we avoided considerations of residual confounding from seafood benefits. Also, in regard to the three cut-off points, recent research [22,24] reflects the occurrence of adverse effects close to the lowest cut-off level. However, it is unclear when effects become negligible, and this uncertainty in particular affects the estimates based on the log curve.

While the use of biomonitoring data from random samples of the general population is an advantage, our calculations were based on sample sizes ranging between 126 and 503 at national and regional levels. The representativeness of the study samples can be challenged, and larger surveys are needed to obtain more precise data, especially in regard to the prevalence of high-level exposures.

Comparison of mean mercury exposure distributions in different countries indicates that, France exhibits Hg concentrations that are greater than in Germany and the US. The consumption of fish in the latter countries is about half that of the French, while countries such as Spain, Sweden and Japan show greater consumption figures [37]. Our results, in line with US findings [33,34], document that a reduction of childhood MeHg exposure may have substantial social benefits. The monetary value of the annual health benefits due to prevention of cognitive disorders generated by a 20% reduction exposure to MeHg in the US population was estimated at $_US_ 170 million/year [29], but this result was certainly underestimated due to underestimation of the hair mercury-IQ dose response slope and the high threshold for neurotoxicity. Using a different approach, Trasande et al. calculated that decreased economic productivity resulting from diminished intelligence over a lifetime results in an aggregate economic cost in each annual birth cohort of $_US_ 8.7 billion annually (range: $0.7–$13.9 billion, $_2000_) [40]. About 15% of this cost was said to be attributable to mercury emitted from coal-fired power plants in the United States [40], although the basis for these calculations may be challenged. In comparing these figures to the ones calculated for France, note should be taken that annual US birth cohorts are about five times greater. Despite the differences in assumptions and published estimates, the results document that benefits of MeHg exposure control are substantial.

Our paper did not estimate the annual costs of investments in pollution abatement because of the paucity of the available data. The known investment costs for Hg emissions control include data from reduction of mercury usage in the chlorine industry (estimation of €_2005_ 0.4 billion), measures taken in dentistry (€_1997_ 0.031 billion), plus expenses for recycling and treatment of mercury releases. These French expenses are total, not annualized. While the utility industry is responsible for a main part of global mercury emissions, its contribution and the costs for abatement vary substantially between countries. In the US, an estimate of $_US_750 million per year has been reported for industrial investments needed to obtain a reduction of Hg emissions [35]. However, abatement efforts should not be undertaken at a national level alone, and calculations need to consider global expenses. Thus, due to regional and hemispherical air dispersion of Hg, a concerted European policy on the emissions is necessary to obtain significant reductions of exposure levels in Europe. The French Institute for Industrial Risks (INERIS) has put forward two main routes for the reduction of mercury releases in the environment: one is substitution of mercury by non-fossils fuels (wood, biogas, biomass) in oil or coal combustion plants and by process changes in the chlorine industry (change to the membrane cell technology); the second would encourage more effective ways to collect and recycle waste containing mercury in batteries, thermometers, dental amalgams, and energy-saving lamps [45]. These costs would have additional socio-economic yields from better control of mercury emissions: job creation and modernization of capital equipment [46].

Methylmercury exposure mainly originates from fish and seafood, which contain essential nutrients that have beneficial effects on brain development [8], [47]. For this reason, a reduction in the concentrations of MeHg in fish is a desirable long-term goal rather than a replacement of fish in the diet by other foods. In the interim, the best method of maintaining fish consumption and minimizing Hg exposure is the consumption of fish known to have lower MeHg concentrations [15] and advisories to women about avoiding Hg intake during pregnancy and breastfeeding may be a cost-effective preventive action.

French studies and recommendations from the French Agency for Food, environmental and Occupational Health Safety (ANSES), stress the need for health education regarding fish species consumption in order to protect vulnerable populations. The INRA study provided evidence on the risks and the benefits of fish consumption by pregnant women to guide decision making in order to reduce risks and optimize nutritional benefits in consumers [3,44]. Thus, implementation of consumption strategies within populations at risk can be in the form of pertinent dietary recommendations [48].

Other factors also play a role in regard to fish consumption. The Sustainable Development indicators show that, despite the implementation of quotas, overfishing continues: 13% of so-called pelagic fish catches (including tuna) are in excess to the precautionary threshold [49] and may for this reason need to be diminished. In this connection, economic aspects of the fishing industry can also be considered. While tuna is high in MeHg content, it is the most popular of seafood species consumed in France (220,000 tons/y between 2008 and 2010) [56, 57], mainly canned (about 94% of the value of household purchases of tuna), and represents 8-9% of household expenditures for fish purchases, i.e. € 0.56 billion in 2010. For comparison, sardines are cheaper and with low MeHg but consumed less frequently (63,000 tons/y), with household expenditures corresponding to € 0.16 billion in 2010. Despite these high figures, the economic importance of high-mercury species is lower than the benefits calculated in the present study, thus emphasizing the need for abatement.

More extensive human biomonitoring would allow a more precise measurement of exposure and would help elaborate recommendations and information to reduce environmental exposures to MeHg [50]. Such studies need to be extended to all of the EU and beyond. However, information alone would not suffice to change dietary habits and taxes and subsidies would be necessary to encourage consumption changes [51]. Our results suggest that the benefits of exposure control justify such actions.

## Conclusions

Annual benefits of removing Hg exposure can be estimated in the order between € 1 billion and € 9 billion in France. While our results support enhanced public policies for the prevention of MeHg exposure, the economic estimates are highly influenced by uncertainties regarding the dose–response relationship. Benefits might be underestimated because costs linked to all aspects of neurotoxicity and to cardiovascular diseases have not been considered. The data from France support the notion that precautionary measures are called for to minimize exposure to this hazardous pollutant.

## Abbreviations

ANSES: French Agency for Food, environmental and Occupational Health Safety; HHg: hair mercury; DRF: Dose–response Function; ENNS: Etude Nationale Nutrition Santé; Hg: Mercury; MeHg: Methylmercury; INERIS: French Institute for Industrial Risks; INRA, National Institute of Agricultural Research; INSERM: National Institute of Health and Medical Research; InVS: French Institute for Public Health Surveillance; IQ: Intelligence Quotient; JECFA: Joint FAO/WHO Expert Committee on Food Additives; NRC: U.S. National Research Council; PÉLAGIE: Longitudinal study on pathologies of pregnancy, infertility and childhood; Pb: Lead; RfD: Reference Dose.

## Competing interests

The authors declare that they have no competing interests.

## Authors’ contributions

CP performed the literature review, drafted the manuscript and carried out the analysis. MB, PG, DZN, NF, SC, ARG and PH contributed substantially to defining the methods of the analysis, interpreting the results of the study and editing the manuscript. All authors read and approved the final version.
